# Development of a questionnaire to assess the medication literacy of patients receiving oral anticancer drugs

**DOI:** 10.1038/s41598-026-46355-7

**Published:** 2026-04-08

**Authors:** Wolfgang Fehrmann, Anna Katharina Moritz, Vanessa Basten, Markus K. Schuler, Matthias Schmid, Nicole Ernstmann, Ulrich Jaehde

**Affiliations:** 1 Department of Clinical Pharmacy, Institute of Pharmacy, University of Bonn, An der Immenburg 4, Bonn, 53121 Germany; 2 Faculty of Medicine and University Hospital Cologne, Chair of Health Services Research, Institute of Medical Sociology, Health Services Research, and Rehabilitation Science (IMVR), University of Cologne, Cologne, Germany; 3 Institute of Medical Biometrics, Informatics, and Epidemiology, University Hospital Bonn, Bonn, Germany; 4 Department of Internal Medicine I, University Hospital Carl Gustav Carus, Technical University at Dresden, Dresden, Germany; 5 Oncology Practice Oskar-Helene-Heim, Berlin, Germany

**Keywords:** Oral anticancer therapy, Medication literacy, Health literacy, Questionnaire development, Cancer, Health care, Medical research, Oncology

## Abstract

Medication literacy is particularly important for patients undergoing oral anticancer therapy, as this treatment involves additional challenges and responsibilities compared to intravenous chemotherapy. The aim of this study was to develop a questionnaire to measure the medication literacy of patients receiving oral anticancer therapy. Following semi-structured interviews and an expert focus group, a first draft of the questionnaire was developed. The draft consisted of two parts, each containing four dimensions. Part A of the questionnaire measured patients’ self-assessed medication literacy, whereas Part B measured their functional medication literacy. After this, both parts of the draft questionnaire were evaluated by conducting a patient survey in oncology practices and community pharmacies. Based on the results of factor analyses, the questionnaire was adapted and finalised. Lastly, the criterion-related validity of the final questionnaire was tested using linear regression analysis, with the aim of investigating its possible influence on quality of life and patient enablement. Factor analysis resulted in a questionnaire that contained 27 items across 7 dimensions. A total of 307 patients was included in the patient survey. Patients achieved an average of 36.7 out of 50 points in Part A and 11.7 out of 17 points in Part B of the final instrument, with higher scores indicating better results. Part A was found to be positively associated with quality of life and patient enablement. While Part A exhibited sound psychometric criteria, Part B still lacked sufficient internal consistency. In conclusion, we developed the first medication literacy questionnaire designed completely from scratch for cancer patients receiving oral anticancer therapy. The part on self-assessed medication literacy could be used as a patient-relevant endpoint or for identifying patients in need of further support with their medication. The part on functional medication literacy needs further refinement. This project is registered in the German Clinical Trials Register (no. DRKS00034341, registration date: 2024-06-03; https://drks.de/search/en/trial/DRKS00034341).

**Supplementary Information:**

The online version contains supplementary material available at 10.1038/s41598-026-46355-7.

## Introduction

Patients receiving oral anticancer drugs are particularly responsible for the success of their therapy^1^. They must be able to understand and evaluate drug-specific information, such as medication plans and package inserts, in order to make informed decisions. These skills are summarised under the term “medication literacy” which is closely related to health literacy. Medication literacy is a relatively new concept, with the first questionnaire designed to measure it, the MedLitRxSE, being published in 2012^2^. Pouliot et al., defined medication literacy through an international Delphi survey with members of the International Pharmaceutical Federation as “the degree to which individuals can obtain, comprehend, communicate, calculate and process patient-specific information about their medications to make informed medication and health decisions in order to safely and effectively use their medications, regardless of the mode by which the content is delivered (e.g. written, oral and visual)”^3^. Furthermore, the survey defined the goals and outcomes of medication literacy, as well as the necessary information and skills for patients to safely take their medication. Patients should, for example, be able to act upon basic medication instructions, calculate the prescribed dose of their medication and make informed decisions regarding their medication and health. While relatively little is currently known about the medication literacy of cancer patients, a considerable amount of research has been conducted on health literacy and cancer. Depending on the study, 11.9 to 86.0% of all cancer patients were found to have limited health literacy, suggesting that a significant proportion of cancer patients show signs of low health literacy^4^. A health literacy survey conducted in Germany found that even in a developed country, almost 73% of all respondents with a chronic illness such as cancer had limited health literacy, with 54% of patients struggling to understand the package inserts of their medicines^5,43^. Health literacy is also especially relevant for cancer patients. Multiple studies have shown, that health literacy affects patient-relevant endpoints such as quality of life in these patients^6^.

Low health literacy and medication literacy could therefore have severe consequences, particularly for patients receiving oral anticancer therapy. Unlike intravenous treatment, which is administered by healthcare professionals, patients receiving oral anticancer therapy must manage their own drugs, which can be challenging if they have low health literacy^7^. Consequently, patients need to be knowledgeable about their medication in order to adhere to it sufficiently^8^. In addition, interactions with oral anticancer drugs are common as many of these drugs have the potential for cytochrome P450 (CYP) interactions with different medications or foods, which can lead to dangerous adverse drug reactions^9,10^. Patients receiving an oral anticancer therapy should therefore be empowered to obtain, understand, and use drug information in order to ensure a safe drug treatment. To our knowledge, only one study to date has specifically addressed medication literacy among cancer patients. In a Chinese cross-sectional study on patients receiving targeted epidermal growth factor receptor tyrosine kinase inhibitors (EGFR-TKIs), 63.5% of all patients were categorised as having “marginal medication literacy” and 22.3% as having “inadequate medication literacy”. A low medication literacy score was found to correlate with a higher proportion of severe skin adverse drug reactions^11^. The study used a translated, therapy-adapted version of the MedLitRxSE to measure medication literacy^2,12^. The MedLitRxSE, however, focuses solely on functional medication literacy, meaning other important medication literacy skills defined by Pouliot et al., such as communication, are missing. Although other tools have been developed from scratch to measure medication literacy in specific patient groups, such as those with diabetes mellitus, hypertension and elderly patients, none have focused on oral anticancer therapy^13–15^. Our goal was therefore to develop a German-language questionnaire, following the definition by Pouliot et al., that measures the medication literacy of patients undergoing oral anticancer therapy and to make initial statements about the validity of the instrument.

## Methods

### Design and setting

The research project “Development and validation of a questionnaire to measure medication literacy in patients receiving oral anticancer drugs” (AMIKO) aimed at developing a questionnaire that would measure patients’ medication literacy when undergoing oral anticancer therapy. The project ran from February 2023 to January 2025. Informed consent was obtained from all participants. All phases were performed in compliance with relevant laws and institutional guidelines. This project is registered in the German Clinical Trials Register (no. DRKS00034341, registration date: 2024-06-03; https://drks.de/search/en/trial/DRKS00034341).

### Development of a draft questionnaire

A systematic review by Gentizon et al.^16^ was used to identify pre-existing relevant medication literacy items and questionnaires. The AMIKO project group, which consisted of an oncologist, two pharmacists, two health services researchers, and a patient representative, used this information to develop an initial draft questionnaire. The content of the questionnaire and its presumed dimensions were based on the defined necessary skills and outcomes of medication literacy defined by Pouliot et al.^3^. When developing the dimensions, the project group also drew on the definitions of general health literacy by Nutbeam^17^ and Sørensen et al.^18^. The latter divides health literacy into three categories: basic/functional literacy, communicative/interactive literacy and critical literacy. The project group decided to add the third category, originally defined as “more advanced cognitive skills which, together with social skills, can be applied to critically analyse information, and to use this information to exert greater control over life events and situations.”, as an additional dimension to the questionnaire as this aspect of health literacy was not considered by Pouliot et al.^3^. The eight defined dimensions with descriptions are shown in Table 1.

**Table 1:** Dimensions of medication literacy defined for the draft questionnaire, along with their respective definitions^3,17,18^.

Dimension	Definition
Obtain	The ability to obtain further information about drugs
Communicate	The ability to communicate drug-related questions or problems to healthcare professionals
Understand	The ability to understand drug information and apply it to one’s own situation
Appraise	The ability of the patient to appraise and assess drug information
Make Decisions	The ability to make informed decisions about medications based on drug information
Calculate	The ability to independently calculate drug dosages based on a medication plan
Contact	The ability to assess when symptoms indicate an emergency and whom to contact
Critically analyse	Advanced cognitive and social skills used to critically analyse drug information and use it to improve one’s own situation and that of other patients

For the first part of the questionnaire (Part A), which was designed to measure patients’ self-assessed medication literacy, items were formulated using a Likert scale with five response options. Part B of the questionnaire was designed to measure patients’ functional medication literacy. For this purpose, single-choice questions with five different response options and a dichotomous scoring were developed.

A qualitative study design was applied to support content validity, consistent with COSMIN recommendations^19^. To further develop the questionnaire, 21 semi-structured interviews were conducted with patients undergoing oral anticancer therapy and, where applicable, their relatives between June 2023 and January 2024. The interviews aimed to explore patient-relevant experiences and challenges related to oral anticancer therapy in order to identify potentially overlooked aspects, particularly with regard to the relevance and comprehensiveness of the predefined dimensions and items. Participants were recruited through two German outpatient oncology practices. Both practices treat patients with solid and haematological malignancies and routinely administer oral anticancer therapies. Eligible participants were adults (≥ 18 years) with a confirmed cancer diagnosis (selected ICD-10 codes) who were currently receiving at least one oral anticancer agent, managed their medication independently, and had provided written informed consent. Exclusion criteria included insufficient German language skills, conditions preventing participation in an interview (e.g. dementia), off-label anticancer therapy, or lack of written consent. To capture a broad range of patient perspectives, purposeful sampling with the aim of maximum variation was applied. Sampling criteria were predefined and included age, gender, educational level, diagnosis, class of oral anticancer therapy, duration of treatment, and prior experience with oral anticancer therapy. Potential participants were informed about the study in the waiting area of the participating practices and referred to the study team if interested. Subsequently, sociodemographic and treatment-related characteristics were assessed in a telephone screening to ensure inclusion of a heterogeneous sample reflecting diverse experiences with oral anticancer therapy. The interviews were guided by the predefined conceptual framework based on the medication literacy model proposed by Pouliot et al.^3^, as well as the general definitions of health literacy by Nutbeam^17^ and Sørensen et al.^18^. Interview questions were aligned with these predefined dimensions of medication literacy to elicit patient-relevant challenges and experiences related to oral anticancer therapy. The interview guide was developed by the project group in a multi-stage, theory-driven process. In a first step, numerous potential questions were generated in an open brainstorming session, based on the dimensions of medication literacy (e.g., obtaining, understanding, and appraising information). In a second step, the questions were critically reviewed with regard to their suitability for answering the research question. Unsuitable and redundant questions were deleted. The remaining questions were then grouped by topic and transferred into a logically structured guideline. To optimise the guide, a test interview was conducted, the results of which were incorporated into the final revision. An excerpt of the interview guide is provided in Supplement S1. The telephone interviews were audio recorded. The total duration of the audio material was 12 h and 50 min (average length: 37 min; range: 18–63 min). Fifteen women and six men were interviewed, with an average age of 69.6 years (SD = 15.1; range = 28–86). Eight patients had a university entrance qualification, two were employed, and twelve lived alone. The sample included patients with solid and blood cancers, twelve were receiving oral anticancer therapy for the first time. The duration of treatment ranged from less than one year to up to seven years. The interviews were professionally transcribed and a structured content analysis according to Kuckartz and Rädiker^20^ was conducted using MAXQDA 2022. The predefined dimensions of medication literacy (see Table 1) served as a priori categories guiding the qualitative analysis. In addition, an online focus group interview was conducted with six experts from different professions (two oncologists, two nurses and two pharmacists) and three patient representatives. Experts were recruited through internet-based searches and recommendations from clinical practitioners. Potential participants were invited via email. Inclusion criteria for experts comprised a minimum of ten years of professional experience and regular involvement in the information provision and education of patients receiving oral anticancer therapy. The focus group discussion was conducted over approximately three hours and was facilitated by members of the project group using a semi-structured guide (see Supplement S2 for selected guiding questions). Two members of the project group (a pharmacist and a health service researcher) developed the guide and its questions, which were then critically reviewed by the other members. During the session, the dimensions and selected questionnaire items were systematically discussed and evaluated with regard to their relevance and appropriateness for the target population. Problem areas not previously covered in the questionnaire were added as items or modified accordingly. As part of a cognitive pre-test, the preliminary questionnaire was tested on five laypersons. The think-aloud method was used to identify any comprehension difficulties when completing the questionnaire. The questionnaire was then revised iteratively until no further comprehension difficulties arose.

### Patient survey

The patient survey was conducted between June 2024 and January 2025. With support of the Scientific Institute of Office-Based Haematologists and Oncologists (WINHO), six oncology practices throughout Germany were recruited for the project. Furthermore, the project group enrolled six German community pharmacies . All six oncology practices and two community pharmacies recruited patients for the survey. Inclusion criteria were: (1) an ICD-10 diagnosis of C00-96, D45, D46, D47, D48 or E24.1; (2) the intake of an oral anticancer therapy; (3) an independent drug intake; (4) an age of at least 18 years; and (5) written informed consent to participate in the study. Exclusion criteria were: (1) an off-label anticancer therapy; (2) a lack of German language skills, and (3) other conditions or diseases that would prevent completion of the questionnaire (e.g., dementia).

After providing written informed consent to participate in the study, patients could complete the questionnaire using the online questionnaire tool “SoSci Survey”. Alternatively, patients also had the option of completing the questionnaire in paper form and sending it to the study centre in Bonn. The patient survey was conducted pseudonymously. At least 300 patients were to be recruited in order to ensure a sufficient number of participants for an exploratory factor analysis (EFA)^21^.

### Psychometric evaluation

All analyses were performed using the statistical software R version 4.1.1.^22^. Both parts of the questionnaire were examined using an EFA. This involved maximum likelihood factor analysis with the WLSMV (weighted least squares with mean and variance adjustment) estimator and varimax rotation, performed with the R function *efa()* from the *lavaan* package^23^. Missing values were supplemented using single imputation for all analyses^24^. Proportional odds regression and logistic regression were used for the imputation of ordinal data and dichotomous data, respectively, using the remaining variables as covariates. Items that loaded < 0.5 on the respective factor or had ≥ 0.32 cross-loading were removed^44^. The number of factors to be used was determined using a scree test and the Kaiser criterion. If an EFA was not successful due to a Kaiser-Meyer-Olkin (KMO) coefficient that was too low (< 0.50)^25^, the originally assumed factor structure was tested using a confirmatory factor analysis (CFA) to assess the extent to which the model fitted the collected data. The original factor structure included the assumed dimensions with the corresponding items and correlations between the individual factors, but not between the items. The CFA was performed using the *cfa()* function from the R package *lavaan*^23^. The tested model was evaluated using the *p*-value of the chi-square test and the goodness-of-fit indices RMSEA (Root Mean Squared Error of Approximation), CFI (Comparative Fit Index), TLI (Tucker-Lewis Index), SRMR (Standardized Root Mean Squared Residual), and WRMR (Weighted Root Mean Square Residual). A *p*-value > 0.05, an RMSEA < 0.05, a CFI > 0.95, a TLI > 0.95, an SRMR < 0.1, and a WRMR < 1 indicated a good model fit^26^. If the goodness-of-fit indices indicated that the model did not fit well, modification indices were used to evaluate possible model improvements. A high modification index indicated that the corresponding adjustment of the model (e.g., with regard to correlations or shifts of items) could lead to a significant improvement in model fit^27^. When selecting model adjustments, the content plausibility was always taken into account alongside the modification index (e.g. with regard to correlations or shifts of items).

The criterion-related validity of the final questionnaire was determined by evaluating the influence of parts A and B on patient enablement and quality of life via univariable linear regression analysis. Patient enablement was selected as an external criterion for criterion-related predictive validity because medication literacy is intended to capture patients’ capacity to obtain, understand, and use medication information in ways that support effective self-management which might lead to better health-related quality of life in oral anticancer therapy. This choice is consistent with empirical literature linking literacy-related constructs with patient engagement/activation and self-management outcomes^28,29^. Patient enablement was measured using two modified items of the Patient Enablement Instrument (PEI), developed and validated by Howie et al., with the two items chosen focusing on the patient’s ability to understand and cope with his illness^30^. Both questions are answered using a five-point Likert scale, whereby a score of 1 to 5 points can be achieved for each question. This means that patients can achieve a minimum of 2 points and a maximum of 10 points in this questionnaire^30^. Quality of life was measured using the German Version EQ-5D-5 L-Instrument developed and validated by the EuroQoL-group^31^. The questionnaire comprises five questions, each with a five-point Likert scale, which cover the following dimensions relevant to quality of life: mobility, self-care, usual activities, pain/discomfort, and anxiety/depression. The answers selected result in a five-digit code, which is then converted into a summary index using a Germany-specific EQ-5D-5 L value set. This index can range from − 0.661 to 1, with 1 indicating optimal health^32^. Additionally, the questionnaire includes a visual analogue scale, which enables patients to rate their quality of life on a scale from 0 to 100. On this scale, 0 represents the worst possible state of health, while 100 represents the best. The results of the summary index and the visual analogue scale are evaluated separately. In the regression analyses, the scores for Parts A and B acted as the independent variables, while the total scores for the PEI, the EQ-5D-5 L index, and the EQ-5D-5 L visual analogue scale acted as the dependent variables. The reliability of the final dimensions was determined using McDonald’s Omega (ω) as a measure for internal consistency based on the results of the EFA and CFA, respectively. Omega values were regarded as acceptable, when they reached a value above 0.7^33,34^.

### Use of large language models

During the preparation of this work the authors used DeepL Write and DeepL Translate in order to translate the questionnaire and improve the readability and language of this article. After using these services, the authors reviewed and edited the content as needed and take full responsibility for the content of the publication.

## Results

### Development of the draft questionnaire

Based on the literature review, semi-structured interviews and focus group interviews, an initial draft questionnaire consisting of 51 items was developed. Part A of the questionnaire consisted of the dimensions *Obtain*, *Communicate* and *Critically analyse* and contained a total of 23 items. Part B consisted of 28 items and contained the dimensions *Appraise*, *Calculate*,* Contact*,* Make decisions* and *Understand*. The subsequent cognitive pre-test involved a total of five laypersons, during which a total of 16 items were removed from the questionnaire and three new items were added. Of the remaining 35 items, 31 items were modified to varying degrees. The most relevant changes included moving the *Appraise* dimension and its items from Part B to Part A and adding an explanatory introductory text to Part B to inform patients how to deal with items relating to situations they had not yet experienced. The final draft questionnaire consisted of a total of 38 items, with 21 items assigned to Part A and 17 items to Part B. It took the participants around 30 min to complete this preliminary questionnaire. A summary of all the items included after the pretest, along with their wording, can be found in Supplement S3.

### Patient characteristics

A total of 359 patients initially agreed to be included in the evaluation, of whom 307 (85.5%) were considered for the final evaluation. The main reason for exclusion was the complete absence of a questionnaire, followed by an incomplete declaration of consent and the withdrawal of consent by the participant. Three patients were excluded because they were receiving off-label therapy and thus met one of the exclusion criteria for the patient survey. A flow chart detailing the process can be found in Supplement S4. The majority of patients were recruited in oncology practices (301; 98.0%) and only a small proportion in community pharmacies (6; 2.0%). A further description of the study population can be found in Table 2. Due to the sometimes inaccurate self-reporting of the patients’ cancer and the associated difficulty in distinguishing between the original cancer and any metastases that might have been present, it was decided not to further distinguish between cancer types.

**Table 2:** Basic characteristics of the participants in the patient survey (*n* = 307).

Variable		
Age in years^a^	Mean value	66.4
	SD [range]	11.9 [27-94]
Sex^a^	Female, *n* (%)	184 (60.9)
	Male, *n* (%)	118 (39.1)
Type of cancer	Haematological, *n* (%)	184 (59.9)
	Solid, *n* (%)	123 (40.1)
Number of oral anticancer drugs	Mean value	1.13
	SD [range]	0.34 [1-3]
Months since initial diagnosis^b^	Mean value	76.7
	SD [range]	77.9 [0-398]
Months since first intake of oral anticancer drug^c^	Mean value	48.1
	SD [range]	65.7 [0-394]
Current living situation	Alone, n (%)	87 (28.3)
	With partner, n (%)	173 (56.4)
	Nursing home / assisted living, n (%)	1 (0.3)
	With family, n (%)	41 (13.4)
	Other, n (%)	5 (1.6)
Monthly household income^d^	Less than € 1,000, n (%)	11 (3.8)
	Between € 1,000 and less than € 2,000, n (%)	63 (21.8)
	Between € 2,000 and less than € 3,500, n (%)	105 (36.3)
	Between € 3,500 and less than € 5,000, n (%)	66 (22.8)
	Between €5,000 and less than €7,000, n (%)	33 (11.4)
	Between €7,000 and less than €10,000, n (%)	9 (3.1)
	€ 10,000 or more, n (%)	2 (0.7)
Highest educational qualification^e^	Primary school leaving certificate, n (%)	18 (5.9)
	Secondary school leaving certificate, n (%)	27 (8.8)
	Intermediate school leaving certificate, n (%)	80 (26.1)
	Journeyman’s certificate, n (%)	24 (7.8)
	A-levels, n (%)	32 (10.5)
	Master craftsman’s certificate, n (%)	10 (3.3)
	Polytechnic degree, n (%)	41 (13.4)
	University degree, n (%)	62 (20.3)
	Higher university degree (e.g. PhD), n (%)	12 (3.9)

^a^Information was available for 302 patients;

^b^Information was available for 292 patients;

^c^Information was available for 291 patients;

^d^Information was available for 289 patients;

^e^Information was available for 306 patients.

### Results of the draft questionnaire

Patients achieved an average of 80.23 (SD = 13.25) out of 105 possible points (76.4%) in Part A and 11.73 (SD = 2.47) out of 17 possible points (69.0%) in Part B. An overview of the items, including difficulty, item discrimination and missing answers, can be found in Table 3. The two items from the PEI had an average value of 8.61 (SD = 1.34). The EQ-5D-5 L index and visual analogue scale had average values of 0.85 (SD = 0.17) and 69.87 (SD = 18.64), respectively.

**Table 3:** Descriptive results of the draft questionnaire (*n* = 307).

Item number	Dimension	Mean (SD)	Difficulty^a^	Item discrimination^b^	Missing answers (%)
Part A					
1	Obtain	4.30 (0.86)	0.82	0.66	1 (0.3%)
2	Obtain	4.13 (0.96)	0.78	0.64	1 (0.3%)
3	Obtain	3.40 (1.27)	0.60	0.60	4 (1.3%)
4	Obtain	3.27 (1.32)	0.57	0.55	5 (1.6%)
5	Communicate	4.16 (0.97)	0.79	0.66	3 (1.0%)
6	Communicate	3.96 (1.14)	0.74	0.63	2 (0.7%)
7	Communicate	4.36 (0.85)	0.84	0.72	3 (1.0%)
8	Communicate	3.92 (1.04)	0.73	0.65	2 (0.7%)
9	Critically analyse	2.89 (1.43)	0.47	0.52	4 (1.3%)
10	Critically analyse	4.40 (0.86)	0.85	0.58	0 (0.0%)
11	Critically analyse	4.21 (0.95)	0.80	0.59	1 (0.3%)
12	Critically analyse	4.02 (1.15)	0.75	0.66	2 (0.7%)
13	Critically analyse	3.39 (1.32)	0.60	0.43	1 (0.3%)
14	Critically analyse	3.84 (1.24)	0.71	0.45	2 (0.7%)
15	Critically analyse	4.26 (1.04)	0.81	0.58	2 (0.7%)
16	Communicate	4.00 (1.07)	0.75	0.56	1 (0.3%)
17	Critically analyse	4.08 (1.21)	0.77	0.28	0 (0.0%)
18	Communicate	4.28 (1.08)	0.82	0.49	3 (1.0%)
19	Appraise	3.10 (1.23)	0.53	0.53	1 (0.3%)
20	Appraise	2.89 (1.36)	0.47	0.50	6 (2.0%)
21	Appraise	3.37 (1.44)	0.59	0.53	2 (0.7%)
Total		80.23 (13.25)			46 (0.7%)^c^
Part B					
22	Make decisions	0.84 (0.37)	0.84	0.35	5 (1.6%)
23	Calculate	0.80 (0.40)	0.80	0.28	3 (1.0%)
24	Contact	0.71 (0.45)	0.71	0.57	6 (2.0%)
25	Understand	0.18 (0.38)	0.18	0.38	9 (2.9%)
26	Make decisions	0.26 (0.44)	0.26	0.13	17 (5.5%)
27	Make decisions	0.34 (0.47)	0.34	0.23	16 (5.2%)
28	Contact	0.82 (0.38)	0.82	0.19	5 (1.6%)
29	Understand	0.90 (0.31)	0.90	0.49	2 (0.7%)
30	Make decisions	0.75 (0.43)	0.75	0.33	12 (3.9%)
31	Calculate	0.94 (0.24)	0.94	0.40	3 (1.0%)
32	Calculate	0.95 (0.22)	0.95	0.52	4 (1.3%)
33	Understand	0.50 (0.50)	0.50	0.53	18 (5.9%)
34	Understand	0.91 (0.29)	0.91	0.76	9 (7.8%)
35	Contact	0.46 (0.50)	0.46	0.18	9 (2.9%)
36	Calculate	0.93 (0.26)	0.93	0.45	5 (1.6%)
37	Contact	0.93 (0.25)	0.93	0.28	3 (1.0%)
38	Understand	0.50 (0.50)	0.50	0.60	18 (5.9%)
Total		11.73 (2.47)			144 (2.8%)^d^

^a^The ratio of points achieved to the total possible points for the respective item.

^b^Part-whole-corrected selectivity.

^c^Out of 6447 possible answers.

^d^Out of 5219 possible answers.

### Factor analyses

An EFA was performed for both parts of the questionnaire. For Part A, the KMO coefficient yielded a value of 0.85 indicating that the dataset was suitable for an EFA. The analysis yielded three factors for Part A of the questionnaire. A total of eleven items were removed due to low primary loadings or high cross loadings, so that a total of ten items remained in Part A of the questionnaire after factor analysis. After analysing the remaining items, the resulting factors corresponded to the specified dimensions *Obtain*, *Communicate* and *Appraise*. The remaining items of the *Critically analyse* dimension were included in the *Communicate* dimension. An overview of the results of the EFA can be found in Table 4.

**Table 4:** Factor loadings of the final EFA model for Part A (*n* = 307).

	Obtain	Communicate	Appraise
Item 02	**0.70**	0.21	0.24
Item 03	**0.75**	0.22	0.18
Item 04	**0.62**	0.16	0.25
Item 11	0.21	**0.73**	-0.04
Item 12	0.30	**0.68**	0.18
Item 16	0.11	**0.76**	0.09
Item 18	0.10	**0.61**	0.18
Item 19	0.22	0.09	**0.84**
Item 20	0.21	0.08	**0.95**
Item 21	0.30	0.19	**0.67**

The primary factor loadings are shown in bold.

**Fig. 1:**
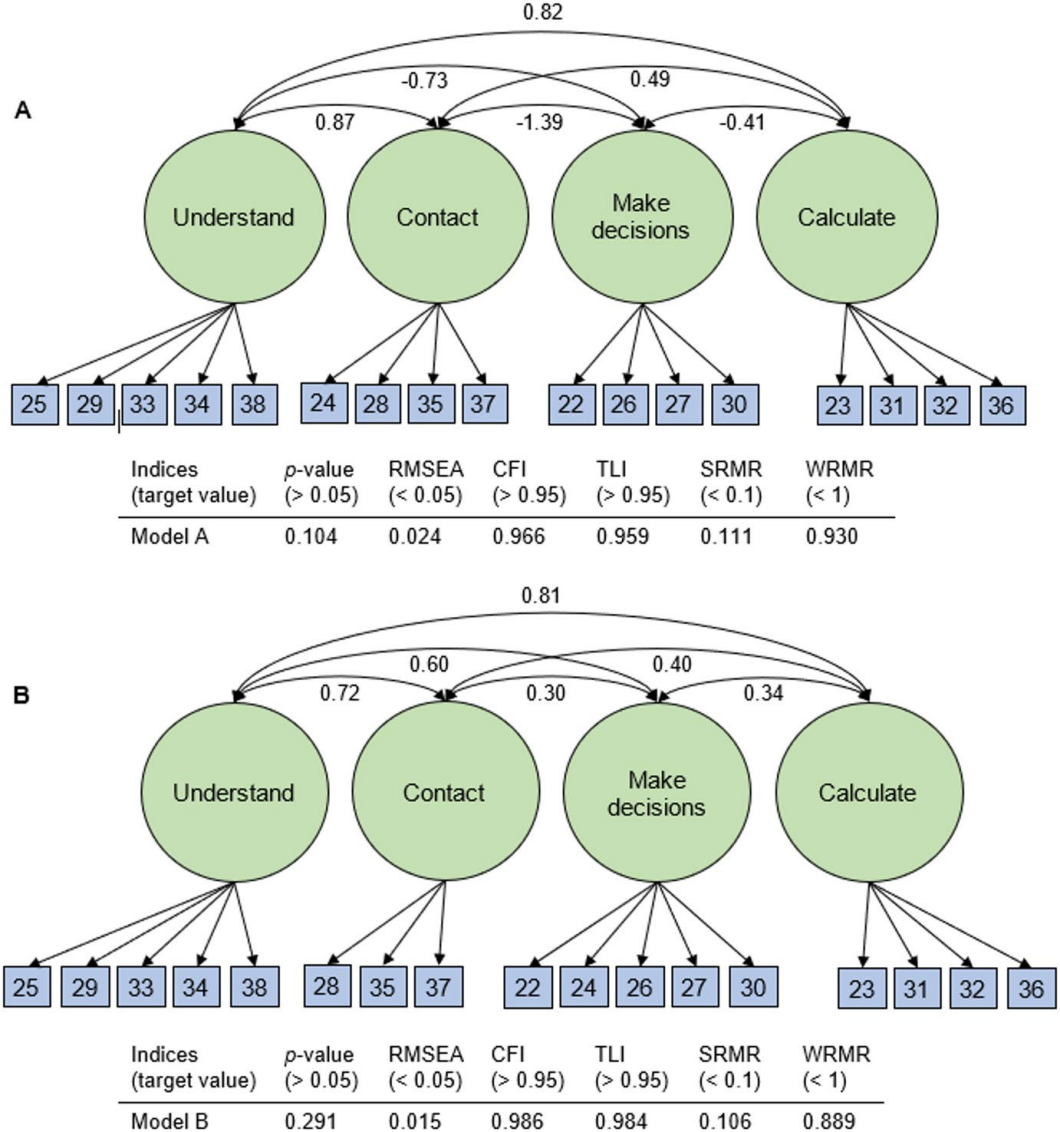
Results of the CFA for Part B. Path diagram and goodness-of-fit-indices for the original model (A) and the model after item 24 was shifted (B) (*n* = 307). Single-headed arrows signify causal relationships and double-headed arrows signify correlations. Each item is represented by its corresponding item number.

For Part B, the KMO criterion of 0.06 indicated that the sample was not suitable for performing an EFA. Instead, the factor structure of Part B was examined using CFA. This factor analysis was based on the presumed factor structure consisting of the four dimensions: *Understand*, *Make decisions*, *Contact* and *Calculate*. The results showed that the goodness-of-fit indices, with the exception of the SRMR, achieved acceptable values (see Fig. 1)^26^.

Following an examination of the modification indices, item 24 from the *Contact* dimension was moved to the *Making Decisions* dimension, which resulted in an improvement in all goodness-of-fit indices. However, the SRMR remained just above the specified limit of 0.1, at 0.106. The primary factor loadings of the models for Part B of the questionnaire are described in Table 5. Although we observed high correlations between *Understand* and *Contact* and *Understand* and *Calculate* in the CFA, these dimensions were not consolidated as the items were considered too dissimilar.

**Table 5:** CFA factor loadings for Part B before and after moving item 24.

	Before moving item 24		After moving item 24	
	Dimension	Factor loadings	Dimension	Factor loadings
Item 22	Make decisions	-0.49	Make decisions	0.49
Item 23	Calculate	0.49	Calculate	0.49
Item 24	Contact	0.66	Make decisions	0.91
Item 25	Understand	0.44	Understand	0.44
Item 26	Make decisions	-0.22	Make decisions	0.26
Item 27	Make decisions	-0.37	Make decisions	0.43
Item 28	Contact	0.22	Contact	0.36
Item 29	Understand	0.60	Understand	0.60
Item 30	Make decisions	-0.44	Make decisions	0.48
Item 31	Calculate	0.62	Calculate	0.62
Item 32	Calculate	0.75	Calculate	0.76
Item 33	Understand	0.58	Understand	0.58
Item 34	Understand	0.90	Understand	0.90
Item 35	Contact	0.20	Contact	0.34
Item 36	Calculate	0.62	Calculate	0.63
Item 37	Contact	0.27	Contact	0.47
Item 38	Understand	0.68	Understand	0.68

### Final questionnaire and criterion-related validity

The final questionnaire consisted of 27 items and is reported in Supplement S5. Patients achieved an average of 36.7 (73.4%) out of a possible 50 points in Part A and 11.7 (69.0%) out of a possible 17 points in Part B of the final instrument. On average, the highest scores were achieved in the dimensions *Calculate* and *Communicate* while the scores in the dimensions *Make decisions* and *Appraise* were the lowest. An overview of the results for the individual dimensions can be found in Supplement S6. The internal consistency of the dimensions of Part A was deemed acceptable exhibiting McDonald´s Omega values of 0.80 (*Obtain*), 0.86 (*Communicate*) and 0.90 (*Appraise*). However, the internal consistency of Part B’s dimensions was poor with Omega values of 0.56, 0.49, 0.17 and 0.41 for the dimensions *Understand*,* Make decisions*,* Contact* and *Calculate*, respectively. Part A of the questionnaire was significantly associated with both patient enablement (*P* < 0.001) and quality of life as measured by the questionnaire (*P* = 0.007) and the visual analogue scale (*P* = 0.009) at a significance level α = 5%, in the univariate linear regression analyses. R^2^ values were 0.105 and 0.020, respectively. No associations were found for Part B.

## Discussion

In this study we developed and partially validated a questionnaire that measures the medication literacy of patients undergoing oral anticancer therapy. Following factor analysis, the final questionnaire consists of a total of 27 items divided into 7 different dimensions. Part A measures self-assessed medication literacy using a Likert scale and consists of 10 items and the 3 dimensions *Obtain*, *Communicate* and *Appraise*, while Part B measures functional medication literacy using a dichotomous performance test consisting of 17 items and the dimensions *Calculate*, *Understand*, *Contact* and *Make decisions*.

In Part A, a questionnaire consisting of three dimensions was generated demonstrating acceptable internal consistency. This contrasts with Part B, where the originally intended EFA could not be carried out due to the low KMO criterion. One possible reason for this is the relatively small number of items in each dimension (four to five items per dimension), which also exhibit varying levels of difficulty and put the patients in a variety of different therapy situations. This heterogeneity could also explain the unacceptable internal consistency of the dimensions in this part of the questionnaire. While the alternative CFA of Part B mostly confirmed the specified factor structure, the SRMR was slightly outside the acceptable range indicating that the model does not yet describe our dataset satisfactorily^26^.

Using simple linear regression, we identified two patient-relevant endpoints that could be influenced by medication literacy: quality of life and patient enablement. However, it should be noted that the effect could only be observed for Part A of the questionnaire and that the coefficient of determination was low in both cases. Further research is therefore necessary to establish whether these effects can be replicated in further studies and whether they have any clinical relevance.

Although there is limited data on medication literacy in Europe, a few comparisons can be made. For example, in a study conducted by Plaza-Zamora et al.^35^, the medication literacy of clients of Spanish community pharmacies was evaluated, with patients scoring an average of 10.3 out of a possible 14 points, equalling 73.6% of the maximum score. Another study of hospitalised patients aged 65 + in Belgium found that the participants scored an average of 2.9 out of a possible 4 points on each item of the instrument used (72.5% of the maximum score)^36^. Our questionnaire produced similar results, with patients scoring an average of 73.3% of the maximum possible points in Part A and 69.0% in Part B of the final instrument. However, in the only other study in patients undergoing oral anticancer therapy, which was conducted on a population of Chinese patients receiving EGFR-TKIs, participants achieved noticeably worse scores. On average, respondents achieved 6.54 out of a possible 14 points in this questionnaire, representing just 46.7% of the maximum total score^11^. It stands to reason that medication literacy similar to health literacy, varies from country to country^37^. Studies on medication literacy in different Chinese populations mostly show a lower average medication literacy across the board, going down as low as 35.4% of the maximum achievable points in patients with coronary heart disease^38–41^. However, it should be noted that research within the field of medication literacy is still in its infancy. Varying definitions and questionnaire designs have been published, meaning that the results of our questionnaire may not always be comparable to those of other studies^15,16,42^.

One strength of our questionnaire is that its two separate parts measure both patients’ self-assessed and functional medication literacy. Our questionnaire thus combines two different approaches to measuring medication literacy, enabling a more detailed analysis of patients’ medication literacy compared to questionnaires that only measure functional medication literacy such as the MedLitRxSE^2^. Because the two parts of the questionnaire were tested separately, they can be used independently from one another, which allows for a shorter questionnaire if only specific dimensions of medication literacy are to be investigated. Thanks to our multicentre approach, which covers various regions of Germany, the results of our survey might be generalised and applied to patients receiving oral anticancer therapy in different settings and regions. Another advantage of our study is that, by involving various health professionals and patient representatives, we were able to consider different perspectives on medication literacy when developing the questionnaire. At the same time, our questionnaire is the first German-language tool for measuring medication literacy, thus offering a new approach to the hitherto very underrepresented research on medication literacy in Europe. It is also the first tool that has been developed from scratch specifically for cancer patients.

There are also several limitations of our study. First, the test-retest reliability could not be assessed due to the cross-sectional survey. Sensitivity to change should be investigated in further research as this psychometric property is strongly needed to use our instrument as an endpoint in interventional studies. Although we classify oral anticancer drugs as a separate group due to the challenges they present, this class comprises many different drug types with often different interactions and side effects^10^. This fact was considered when developing the items by selecting problems and tasks that were applicable to as many different drug types as possible. Nevertheless, some of the tasks included may not apply to every patient’s oral anticancer therapy. Since the originally planned EFA was not possible, Part B of the questionnaire could only be evaluated using CFA. Furthermore, moving an item from one dimension to another using modification indices carries the risk that the observed effects are only based on sample-specific optimisation. Due to this, and the low internal consistency observed across this section, Part B and its factor structure should be considered exploratory. Further re-examination and addition of items, together with a new factor analysis, are necessary before this part of the questionnaire can be used in patient care. Part A, was only examined using EFA. Further confirmation of the factor structure of both parts of the questionnaire requires an additional CFA to be conducted with a different patient group. As the questionnaire was completed by the patients themselves without medical or pharmaceutical supervision, patients may have received assistance in completing Part B of the questionnaire.

## Conclusions

We developed a questionnaire for measuring the medication literacy of patients receiving oral anticancer therapy. Initial analyses of the questionnaire’s validity suggest a psychometrically sound model for measuring self-assessed medication literacy. This part of the questionnaire can be used as a possible endpoint in studies developing patient education and to identify patients who would particularly benefit from patient-centred information about their medicines. The part of the questionnaire intended to measure functional medication literacy should undergo further refinement before it is ready to use.

## Supplementary Information

Below is the link to the electronic supplementary material.

Supplementary Material 1

Supplementary Material 2

Supplementary Material 3

Supplementary Material 4

Supplementary Material 5

Supplementary Material 6

## Data Availability

The dataset used and analysed during the current study is available from the corresponding author on reasonable request.
