# Supplementary material for: Development of a questionnaire to assess the medication literacy of patients receiving oral anticancer drugs
Source: Sci Rep. 2026 Apr 8;16:12029. doi: 10.1038/s41598-026-46355-7 (PMC13068952; doi:10.1038/s41598-026-46355-7)
Supplement: Supplementary file 1 — Supplementary Material 1 [file 41598_2026_46355_MOESM1_ESM.pdf]

Supplement S1: Extract from the patient interview guideline
(translated from the original German version)

Main questions	Supporting questions
**1. Initial question / medication intake**<br>*You are currently being treated for cancer and are receiving tumor therapy in tablet or capsule form (called oral tumor therapy). We are interested in how you take your medication at home.*<br>Could you start by briefly telling me the name of the medication you are taking?<br>Can you please tell me exactly how you take your medication?	- What do you do differently from other medications? / What do you pay attention to? *For example: Medication instructions; Storage/disposal; Dealing with excretions; Handling in case of forgetting/vomiting*<br>- How do you handle the medication when you are not at home?
**2. Drug-drug/Drug-food interactions**<br>To what extent do you pay attention to interactions with your cancer medication?	- Are there any foods you need to avoid?<br>- Are there any medications you need to avoid?
**3. Dealing with drug information**<br>*Our project is primarily concerned with drug information. This can be verbal information, but also written information.*<br>How do you deal with this information about your cancer medication?	- Which information do you find particularly difficult to understand?<br>- How do you cope with technical terms?<br>- How do you cope with written information, e.g., the package insert?<br>- What would you change in terms of the information about your medications?<br>- Sometimes patients have to calculate dosages themselves. How do you cope with this?
**4. Medication prescription**<br>Can you tell me how the conversation went when the doctor prescribed your cancer medication?	- To what extent were you informed of other treatment options?<br>- To what extent were you informed about possible adverse events?
**5. Information needs**<br>If you remember the first day you came home with your cancer medication...did you feel well informed and educated?<br>[Alternatively: Did you know the things you need to know when taking your medication?]	- Were you able to apply the information you received in your everyday life?<br>- What information did you miss?<br>- How would you have liked to receive information and advice about your medication?<br>- Are you missing further persons to contact on specific questions?

**6. Searching for and finding drug information**<br>What do you do if you still have questions about your cancer drugs?	- Who do you contact if you have questions about medication?<br>- What kind of support do you seek in your private life if you have questions about medication?<br>- Where do you look for further information if you have questions about medication?<br>- What difficulties do you encounter when using media/the internet?
