# Supplementary material for: Development of a questionnaire to assess the medication literacy of patients receiving oral anticancer drugs
Source: Sci Rep. 2026 Apr 8;16:12029. doi: 10.1038/s41598-026-46355-7 (PMC13068952; doi:10.1038/s41598-026-46355-7)
Supplement: Supplementary file 2 — Supplementary Material 2 [file 41598_2026_46355_MOESM2_ESM.pdf]

Supplement S2: Selected guiding questions from the focus group interview guide

(translated from the original German version)

Topic 3 – Skills and competencies:

Think of a patient from your practice / your personal experiences / members of your support group...

→ In your opinion, what competencies / skills do patients who take oral anticancer therapy at home need?

Topic 4 – Challenges and difficulties:

→ What challenges and difficulties do you see for patients when taking and handling oral anticancer therapy at home?

Topic 5 – AMIKO dimensions and items:

→ In your opinion, how important are the skills shown (dimensions of medication literacy) for your patients?
