# Supplementary material for: Development of a questionnaire to assess the medication literacy of patients receiving oral anticancer drugs
Source: Sci Rep. 2026 Apr 8;16:12029. doi: 10.1038/s41598-026-46355-7 (PMC13068952; doi:10.1038/s41598-026-46355-7)
Supplement: Supplementary file 3 — Supplementary Material 3 [file 41598_2026_46355_MOESM3_ESM.pdf]

Supplement S3: Items included in the patient survey after the pretest

Translated using DeepL Translate

Items included in the patient survey, along with their wording

Item	Dimension	Item wording
**Part A**		
1	Find	I find it easy to find information on the correct intake of my cancer medication.
2	Find	I find it easy to find information about the side effects of my cancer medication.
3	Find	I find it easy to find information about medications I take for the side effects of my cancer treatment
4	Find	I find it easy to find information about dietary supplements or medicines that I buy at the drugstore or pharmacy without a doctor's recommendation.
5	Communicate	I find it easy to clearly communicate to the medical staff what I hope to achieve from my cancer treatment.
6	Communicate	I find it easy to communicate my fears about my cancer treatment to the healthcare professionals in a way they can understand.
7	Communicate	I find it easy to communicate any questions I have about my cancer medication clearly to the healthcare professionals.
8	Communicate	I find it easy to decide which side effects I should inform the healthcare professionals about.
9	Communicate	I try to use my knowledge to help other cancer patients with treatment problems (e.g. acquaintances, fellow patients during an inpatient or outpatient stay, other members of patient organisations).
10	Critically analyse	If I find patient information difficult to understand, I ask healthcare professionals.
11	Critically analyse	When I talk to the healthcare professionals about my cancer treatment and I still have questions, I try to get answers to all of them, regardless of how long it takes.

Item	Dimension	Item wording
12	Critically analyse	If there is anything about my cancer treatment that I do not understand, I will seek support (e.g. from relatives or healthcare professionals).
13	Critically analyse	If there is something about my cancer treatment that I do not understand, I try to find out about it myself (e.g. in patient brochures or on the internet).
14	Critically analyse	When I receive a new anticancer drug, I try to find out exactly how the drug works.
15	Critically analyse	When I look for information about my cancer treatment, I pay attention to who the information comes from.
16	Critically analyse	If I feel overwhelmed by the amount of information about my cancer treatment during a consultation with my doctor, I find it easy to tell them.
17	Critically analyse	When I look for information about my cancer treatment, I pay attention to who the information comes from.
18	Communicate	If I have difficulty taking my medication (e.g. swallowing pills), I find it easy to tell the healthcare professionals.
19	Evaluate	I find it easy to assess whether information about my cancer treatment that I find in an online search is reliable or not.
20	Evaluate	I find it easy to assess whether information about my cancer treatment that I find on social media is reliable or not.
21	Evaluate	I find it easy to assess how I deal with information about my cancer treatment that comes from people who are not in a medical profession (e.g. information from friends or relatives).

Item	Dimension	Item wording
**Part B**		
22	Make decisions	A patient finds the following information in the package insert for his cancer medication. In your opinion, where should he store his tablets that are not currently needed?
23	Calculate	Please look at the medication schedule below.<br>A patient wants to go on vacation for two weeks. How many crizotinib tablets do you think he should take with him at least?
24	Contact	A patient is running out of his cancer medication over the weekend.<br>What should he do in your opinion?
25	Understand	A patient has been prescribed medication for nausea by his doctor. He finds the following information in the package insert.<br>When do you think the patient should take these medications?
26	Make decisions	A patient wants to split his tablets for nausea because they are too large for him and he cannot swallow them whole. He cannot find any information about whether the tablets can be split in the package leaflet.<br>What would you advise him to do?
27	Make decisions	A patient forgot to take his cancer medication yesterday.<br>How should he proceed in your opinion?
28	Contact	A patient has been suffering from a high fever (over 39 °C) for two days, which is listed as a side effect in the package insert for his cancer medication.<br>What would you advise him, should he do?
29	Understand	A patient has been advised that drinking grapefruit juice causes severe interactions with his cancer medication.<br>What does this mean for him in your opinion?
30	Make decisions	A patient suffers from inflammation of the oral mucosa. In a patient information leaflet, he finds the information he should avoid irritation in the mouth area.<br>What do you think he should do?
31	Calculate	A patient takes a medicine twice a day. He has bought 24 tablets at the pharmacy and starts taking them on Monday, 1 July in the morning.<br>On which day of the week do you estimate that he will run out of tablets?

Item	Dimension	Item wording
32	Calculate	A patient has been instructed to take his concomitant medication regularly every 12 hours.<br>When do you think he can take the concomitant medication if the interval between doses is to be as regular as possible?
33	Understand	A patient finds the following information in the package insert for his cancer medication.<br>Which of his medications do you think he should tell his doctor or pharmacist about? Please refer to the patient's medication schedule below.
34	Understand	Take a look at the medication schedule below.<br>At what times of day must the cancer medication be taken according to the medication schedule?
35	Contact	Since starting cancer treatment, a patient has been suffering from mild diarrhoea once a week, which disappears after taking a medically recommended anti-diarrhoea medication. He finds the following information in the patient information leaflet for his cancer medication.<br>In your opinion, how should he proceed?
36	Calculate	A patient takes 140 mg of his cancer medication in the morning.<br>Which tablets do you think are necessary to achieve the desired 140 mg?
37	Contact	A patient is unable to tolerate his current cancer treatment and would therefore like to find out about other possible cancer treatments.<br>In your opinion, who should he contact?
38	Understand	A patient finds the following information in the package insert for a cancer drug.<br>Which statement do you think applies when taking this medication?
