# Supplementary material for: Development of a questionnaire to assess the medication literacy of patients receiving oral anticancer drugs
Source: Sci Rep. 2026 Apr 8;16:12029. doi: 10.1038/s41598-026-46355-7 (PMC13068952; doi:10.1038/s41598-026-46355-7)
Supplement: Supplementary file 4 — Supplementary Material 4 [file 41598_2026_46355_MOESM4_ESM.pdf]

Supplement S4: Patient recruitment for the patient survey

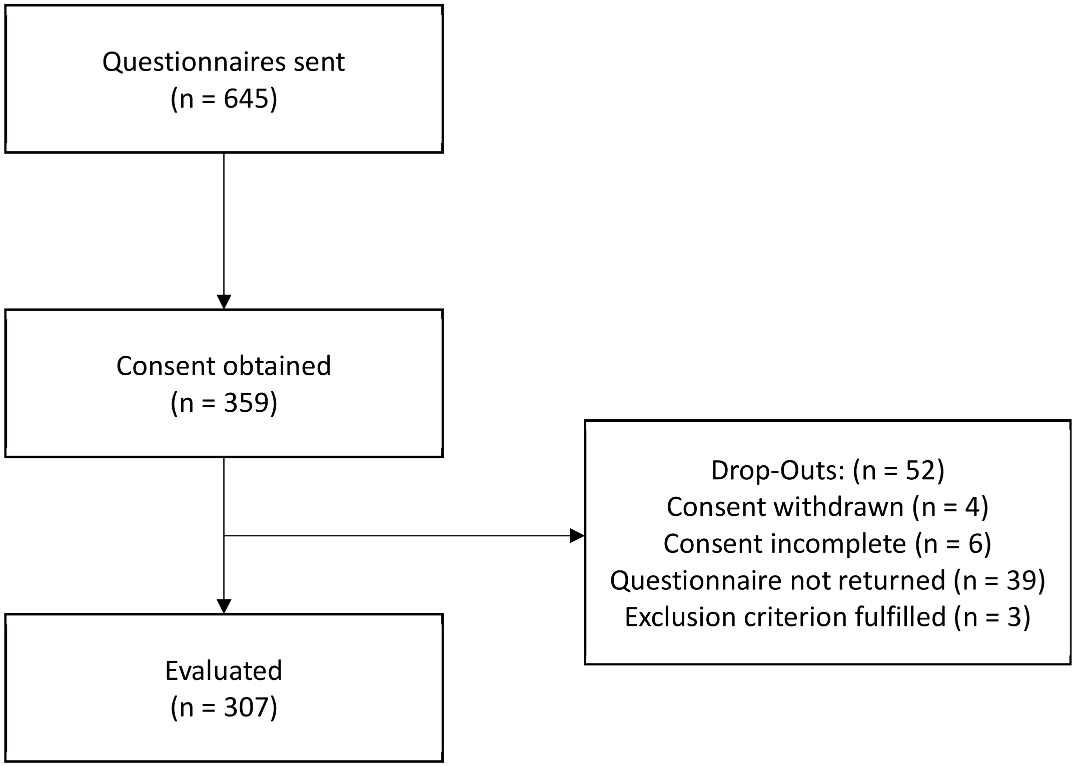

Figure S1 Flow Chart detailing the recruitment for the patient survey
