# Supplementary material for: Development of a questionnaire to assess the medication literacy of patients receiving oral anticancer drugs
Source: Sci Rep. 2026 Apr 8;16:12029. doi: 10.1038/s41598-026-46355-7 (PMC13068952; doi:10.1038/s41598-026-46355-7)
Supplement: Supplementary file 5 — Supplementary Material 5 [file 41598_2026_46355_MOESM5_ESM.pdf]

Supplement S5: Final questionnaire (English translation)

Translated using DeepL Translate

Self-assessment on how you manage your cancer medication

Instructions

Please assess your own abilities in handling medication honestly. For each question, tick the answer that **best applies to you.**

The term "specialist staff" refers to all medical and pharmaceutical professions such as e.g. doctors, nurses, medical assistants and pharmacists.

Some of the following statements may describe situations that have not yet occurred during your treatment. In this case, please give an honest assessment of how you would deal with such a situation if it arose.

Please tick only one answer per question.

	Does not apply at all	Does not really apply	Applies to some extent	Applies to a greater extent	Applies completely
1. I find it easy to find information about the side effects of my cancer medication.	☐	☐	☐	☐	☐
2. I find it easy to find information about medications I take for the side effects of my cancer treatment.	☐	☐	☐	☐	☐
3. I find it easy to find information about dietary supplements or medicines that I buy at the drugstore or pharmacy without a doctor's recommendation.	☐	☐	☐	☐	☐
4. When I talk to the healthcare professionals about my cancer treatment and I still have questions, I try to get answers to all of them, regardless of how long it takes.	☐	☐	☐	☐	☐
5. If there is anything about my cancer treatment that I do not understand, I will seek support (e.g. from relatives or healthcare professionals).	☐	☐	☐	☐	☐
6. If I feel overwhelmed by the amount of information about my cancer treatment during a consultation with my doctor, I find it easy to tell them.	☐	☐	☐	☐	☐
7. If I have difficulty taking my medication (e.g. swallowing pills), I find it easy to tell the healthcare professionals.	☐	☐	☐	☐	☐

	Does not apply at all	Does not really apply	Applies to some extent	Applies to a greater extent	Applies completely
8. I find it easy to assess whether information about my cancer treatment that I find in an online search is reliable or not.	☐	☐	☐	☐	☐
9. I find it easy to assess whether information about my cancer treatment that I find on social media is reliable or not.	☐	☐	☐	☐	☐
10. I find it easy to assess how I deal with information about my cancer treatment that comes from people who are not in a medical profession (e.g. information from friends or relatives).	☐	☐	☐	☐	☐

Your recommendations for the proper use of cancer drugs

Imagine that a patient has cancer. To treat their illness, they receive, like you, a cancer therapy in tablet form from their doctor.

Instructions

Please indicate what you would recommend in the situations described or what decisions the patient should make. For each question, tick the answer you think is correct.
Please tick only one answer for each question!

Please answer the questions in the following section **without the help of other people or aids (internet, package inserts, etc.)**. We are interested in your own assessments of the situations described.

Please note that none of the information in this part of the questionnaire constitutes a recommendation for treatment. Please never adjust your medication yourself without first consulting your doctor!

11. A patient finds the following information in the package insert for his cancer medication. In your opinion, where should he store his tablets that are not currently needed?

Store in a dry place at room temperature.

☐ On the window sill
☐ In the bathroom
☐ In the refrigerator
☐ In a cupboard in the living room
☐ I don't know.

12. Please look at the medication schedule below.
A patient wants to go on vacation for two weeks. How many crizotinib tablets do you think he should take with him at least?

Active Ingredient	Trade name	Strength	Morning	Noon	Evening	Night	Reason
Zopiclon	Ximovan	7,5 mg	0	0	0	1	Sleep disorder
Crizotinib	Xalkori	250 mg	1	0	1	0	Cancer
Metformin	Glucophage	1000 mg	1	0	1	0	Diabetes mellitus
Pantoprazol	Pantozol	40 mg	1	0	0	0	Heartburn

☐ 14 tablets
☐ 20 tablets
☐ 24 tablets
☐ 28 tablets
☐ I don't know.

13. A patient is running out of his cancer medication over the weekend.
What should he do in your opinion?

☐ He can go to the pharmacy and get a new pack of his medication without a prescription.

☐ He should contact his doctor's office or his emergency number and find out what to do next.

☐ He should call the general emergency number (112) and find out what to do next.

☐ He can safely discontinue taking his cancer medication for a weekend.

☐ I don't know.

14. A patient has been prescribed medication for nausea by his doctor. He finds the following information in the package insert.
When do you think the patient should take these medications?

Method of Administration
Swallow the medication with a full glass of water. Do not eat anything for at least 1 hour before taking the medication and for at least 2 hours after taking it.

☐ One hour before a meal

☐ 1.5 hours after a meal

☐ 30 minutes before a meal

☐ Immediately after a meal

☐ I don't know.

15. A patient wants to split his tablets for nausea because they are too large for him and he cannot swallow them whole. He cannot find any information about whether the tablets can be split in the package leaflet.
What would you advise him to do?

☐ The tablets can be split if they have a score line.

☐ He must ask a healthcare professional about the divisibility of his tablets.

☐ If there is no information in the package insert, the tablet can be divided.

☐ Tablets should generally not be split.

☐ I don't know.

16. A patient forgot to take his cancer medication yesterday. How should he proceed in your opinion?

☐ He should take twice the amount of his cancer medication today.

☐ He should take 1.5 times the amount of his cancer medication today.

☐ He should consult his doctor, pharmacist or the package insert for instructions on how to proceed

☐ He should take the usual dose of his cancer medication today.

☐ I don't know.

17. A patient has been suffering from a high fever (over 39 °C) for two days, which is listed as a side effect in the package insert for his cancer medication.
What would you advise him, should he do?

- ☐ Stop taking the medication on his own and only resume taking the tablets once the fever has subsided
- ☐ Continue taking the medicine as usual and inform his doctor at his next appointment.
- ☐ Continue taking the medicine as usual; no further action is necessary.
- ☐ Contact his doctor immediately.
- ☐ I don't know.

18. A patient has been advised that drinking grapefruit juice causes severe interactions with his cancer medication.
What does this mean for him in your opinion?

- ☐ It does not matter whether he drinks grapefruit juice during cancer treatment or not.
- ☐ He can drink grapefruit juice during cancer treatment, but only once a day
- ☐ He should not drink grapefruit juice during cancer treatment.
- ☐ He can continue to drink grapefruit juice, but he should not take it at the same time as his tablets.
- ☐ I don't know.

19. A patient suffers from inflammation of the oral mucosa. In a patient information leaflet, he finds the information he should avoid irritation in the mouth area.
What do you think he should do?

- ☐ He should not brush his teeth more than once a day.
- ☐ He should avoid spicy and very hot food.
- ☐ He should use alcoholic mouthwash every day.
- ☐ He should use a toothbrush that is as hard as possible.
- ☐ I don't know.

20. A patient takes a medicine twice a day. He has bought 24 tablets at the pharmacy and starts taking them on Monday, 1 July in the morning.
On which day of the week do you estimate that he will run out of tablets?

- ☐ Wednesday, 10 July
- ☐ Friday, 12 July
- ☐ Thursday, 18 July
- ☐ Wednesday, 24 July
- ☐ I don't know.

21. A patient has been instructed to take his concomitant medication regularly every 12 hours. When do you think he can take the concomitant medication if the interval between doses is to be as regular as possible?

☐ At 8:00 a.m. and 8:00 p.m.

☐ At 7:00 a.m. and 5:00 p.m.

☐ At 12:00 a.m. and 8:00 p.m.

☐ At 10:00 a.m. and 8:00 p.m.

☐ I don't know.

22. A patient finds the following information in the package insert for his cancer medication. Which of his medications do you think he should tell his doctor or pharmacist about? Please refer to the patient's medication schedule below.

Tell your doctor or pharmacist if you are taking or intend to take any of the following medications:
- *Anticoagulants (medicines that prevent the formation of thromboses or blood clots): e.g. warfarin*
- *Statins (medicines that lower cholesterol levels): e.g. atorvastatin, rosuvastatin*
- *Proton pump inhibitors (medicines that suppress the formation of stomach acid): e.g. omeprazole, pantoprazole*
- *Azole antifungals (medicines used to treat fungal infections): e.g. ketoconazole, voriconazole*

**Active ingredient**	**Trade name**	**Strength**	**Morning**	**Noon**	**Evening**	**Night**	**Reason**
Chlortalidone	Hygroton	25 mg	1	0	0	0	High blood pressure
Bisoprolol	Bisoprolol AL	5 mg	1	0	1	0	High blood pressure
Simvastatin	Simvastatin Hexal	40 mg	0	0	1	0	Elevated blood cholesterol levels
Loperamid	Imodium	2 mg	Bei Bedarf				Diarrhoea in cancer

☐ Chlortalidone

☐ Bisoprolol

☐ Simvastatin

☐ None of the medications listed

☐ I don't know.

23. Take a look at the medication schedule below.
At what times of day must the cancer medication be taken according to the medication schedule?

Active ingredient	Trade name	Strength	Morning	Noon	Evening	Night	Reason
Chlortalidone	Hygroton	25 mg	1	0	0	0	High blood pressure
Bisoprolol	Bisoprolol AL	5 mg	1	0	1	0	High blood pressure
Simvastatin	Simvastatin Hexal	40 mg	0	0	1	0	Elevated blood cholesterol levels
Erlotinib	Tarceva	150 mg	1	0	0	0	Cancer
Loperamid	Imodium	2 mg	Bei Bedarf				Diarrhoea in cancer

☐ In the morning

☐ At noon

☐ In the morning and at noon.

☐ In the morning, at noon and in the evening

☐ I don't know.

24. Since starting cancer treatment, a patient has been suffering from mild diarrhoea once a week, which disappears after taking a medically recommended anti-diarrhoea medication. He finds the following information in the patient information leaflet for his cancer medication.
In your opinion, how should he proceed?

> *The treatment may cause persistent and severe diarrhoea in some cases. If this happens to you, notify your doctor immediately.*

☐ If he has diarrhoea, he should take twice the amount of the medication.

☐ He should inform his doctor about the side effect at his next appointment.

☐ He must call his doctor immediately and inform him about the side effect.

☐ He should take the medication continuously so that the symptoms do not worsen.

☐ I don't know

25. A patient takes 140 mg of his cancer medication in the morning.
Which tablets do you think are necessary to achieve the desired 140 mg?

☐ One tablet containing 80 mg and two containing 20 mg

☐ Two tablets containing 80 mg.

☐ One tablet containing 80 mg, two containing 20 mg and one containing 10 mg

☐ One tablet containing 80 mg and three containing 20 mg

☐ I don't know

26. A patient is unable to tolerate his current cancer treatment and would therefore like to find out about other possible cancer treatments.
In your opinion, who should he contact?

- ☐ His oncologist
- ☐ His oncologist or general practitioner
- ☐ His general practitioner
- ☐ His pharmacist
- ☐ I don't know

27. A patient finds the following information in the package insert for a cancer drug. Which statement do you think applies when taking this medication?

- *Swallow the capsules whole, preferably with a glass of water.*
- *The capsules must not be broken, opened or chewed. If the contents of the capsule come into contact with the skin, wash the skin thoroughly with soap and water immediately*
- *Healthcare professionals, nursing staff and family members must wear disposable gloves when handling the blister packs or capsules. The gloves must then be removed carefully to avoid skin contact and disposed of in a sealable polyethylene plastic bag in accordance with local regulations. Hands must then be washed thoroughly with soap and water*
- *Pregnant women or women who may be pregnant should not handle the blister packs or capsules.*
- *The capsules should be taken at the same time each day if possible.*
- *The capsules can be taken with or without food.*

- ☐ The capsules may be opened if the patient is unable to swallow the capsule whole.
- ☐ The capsules must be taken immediately after a meal.
- ☐ The patient must wear gloves when handling the capsules.
- ☐ Family members must wash their hands after handling the capsules.
- ☐ I don't know
