# Supplementary material for: Development of a questionnaire to assess the medication literacy of patients receiving oral anticancer drugs
Source: Sci Rep. 2026 Apr 8;16:12029. doi: 10.1038/s41598-026-46355-7 (PMC13068952; doi:10.1038/s41598-026-46355-7)
Supplement: Supplementary file 6 — Supplementary Material 6 [file 41598_2026_46355_MOESM6_ESM.pdf]

Supplement S6: Results of the individual medication literacy dimensions in the final questionnaire

Average scores achieved in Part A of the questionnaire (n = 307, missing data were imputed)

Dimension (number of items; possible scores)		Score (%)
**Obtain (3 Items; 3-15 points)**	Mean value	10.8 (71.9)
	SD [range]	2.9 [3-15]
**Communicate (4 Items; 4-20 points)**	Mean value	16.5 (82.5)
	SD [range]	3.2 [7-20]
**Appraise (3 Items; 3-15 points)**	Mean value	9.4 (62.4)
	SD [range]	3.6 [3-15]
**Part A (10 Items; 10-50 points)**	Mean value	36.7 (73.3)
	SD [range]	7.3 [14-50]

Average scores achieved in Part B of the questionnaire (n = 307, missing data were imputed)

Dimension (number of items; possible scores)		Score (%)
**Understand (5 Items; 0-5 points)**	Mean value	3.0 (59.7)
	SD [range]	1.2 [0-5]
**Make decisions (5 Items; 0-5 points)**	Mean value	2.9 (58.1)
	SD [range]	1.2 [0-5]
**Contact (3 Items; 0-3 points)**	Mean value	2.2 (73.3)
	SD [range]	0.7 [0-3]
**Calculate (4 Items; 0-4 points)**	Mean value	3.6 (90.6)
	SD [range]	0.7 [0-4]
**Part B (17 Items; 0-17 points)**	Mean value	11.7 (69.0)
	SD [range]	2.5 [4-17]
